# Prognostic Value of Inflammation and Nutrition-Based Scores in Low-Risk Myelodysplastic Syndrome: A Retrospective Cohort Study

**DOI:** 10.3390/jcm14134751

**Published:** 2025-07-04

**Authors:** Tuba Ersal, Vildan Özkocaman, Sinem Çubukçu, Tuba Güllü Koca, Fazıl Çağrı Hunutlu, Şeyma Yavuz, Ezel Elgün, Gökhan Ocakoğlu, Fahir Özkalemkaş

**Affiliations:** 1 Division of Hematology, Department of Internal Medicine, Faculty of Medicine, Bursa Uludag University, 16059 Bursa, Turkey; vildanoz@uludag.edu.tr (V.Ö.); sinemcubukcu@uludag.edu.tr (S.Ç.); tubagullukoca@uludag.edu.tr (T.G.K.); fazilhunutlu@gmail.com (F.Ç.H.); seymayavuz2011@hotmail.com (Ş.Y.); elgunezel@hotmail.com (E.E.); fahir@uludag.edu.tr (F.Ö.); 2 Department of Biostatistics, Faculty of Medicine, Bursa Uludag University, 16059 Bursa, Turkey; ocakoglu@uludag.edu.tr

**Keywords:** low-risk myelodysplastic syndrome, inflammation, nutrition, mortality, prognostic nutritional index, systemic oxidative stress

## Abstract

**Background/Objectives:** The pathogenesis of Myelodysplastic Syndrome (MDS) is diverse; however, increasing evidence suggests that inflammation and oxidative stress play a significant role in the development and progression of the disease. This study aimed to evaluate the prognostic impact of inflammation, nutritional status, and oxidative stress at diagnosis in patients with low-risk MDS. **Methods:** A retrospective analysis was conducted on 175 newly diagnosed low-risk MDS patients. **Results:** A low Prognostic Nutritional Index (PNI) and a high systemic oxidative stress (SOS) score were independently associated with poorer prognosis (PNI: HR 1.598, 95% CI 1.076–2.372, *p* = 0.02; SOS: HR 1.003, 95% CI 1.001–1.006, *p* = 0.002). The optimal PNI cut-off value for predicting mortality was identified as 47.47. Based on this cut-off, 92 patients had a low PNI score, while 83 patients had a high PNI score. The comparison between these groups revealed a statistically significant difference in median overall survival (OS), with 45.5 months for the low-PNI group and 75.1 months for the high-PNI group (*p* < 0.001). However, PNI was not significantly associated with progression to acute myeloid leukemia (AML) (*p* = 0.668). In the multivariate OS analysis, several factors were identified as independent predictors of prognosis, including a high Revised International Prognostic Scoring System (R-IPSS) score, low PNI, high SOS score, advanced age, male gender, and transformation to AML. **Conclusions:** Together, PNI and SOS may serve as simple, accessible tools to improve risk stratification in low-risk MDS patients.

## 1. Introduction

Myelodysplastic Syndrome (MDS) is a diverse group of clonal disorders marked by dysplastic changes in the bone marrow, ineffective hematopoiesis, peripheral cytopenia, recurrent genetic abnormalities, and the potential to progress to acute myeloid leukemia (AML) [1]. Although MDS pathologies are varied, increasing evidence suggests that dysregulated innate inflammation is a key driver of both disease phenotype and progression, acting through alterations in the hematopoietic and stromal compartments [2,3,4].

The International Prognostic Scoring System (IPSS) [5] and its revised version (R-IPSS) [6] are essential tools for evaluating the prognosis of untreated MDS patients [7]. However, it is also important to consider additional factors, such as comorbidities, when making treatment decisions. The IPSS assesses bone marrow blast percentage, cytogenetic abnormalities, and the presence of cytopenias. The R-IPSS, on the other hand, evaluates cytogenetics, bone marrow blast percentage, hemoglobin level, platelet count, and absolute neutrophil count. While patients with low-risk MDS generally have a milder disease course, those with high-risk MDS may progressively develop signs of bone marrow failure and have a higher likelihood of progressing to acute myeloid leukemia (AML).

Although both the IPSS and R-IPSS provide significant advantages in guiding clinical decision making, they also present certain limitations. The IPSS is constrained by its reliance on cytogenetics and morphology and its exclusion of other relevant clinical parameters. While the IPSS-R represents an improvement, it is more complex and may lack clarity in rare subgroups, with limited discriminatory power among low-risk patients.

The newly developed Molecular International Prognostic Scoring System (IPSS-M) seeks to address these gaps by incorporating molecular mutation data. However, its routine use remains limited, as molecular testing is not yet widely available in all clinical centers.

As a result, there is growing interest in identifying simpler and more accessible prognostic tools. Several studies have explored alternative prognostic indicators, including comorbidities [8], high serum ferritin levels [9], the presence of bone marrow fibrosis [10], monocytosis [11], hypoalbuminemia [12], alanine aminotransferase (ALT) levels [13], platelet-to-lymphocyte ratio (PLR), neutrophil-to-lymphocyte ratio (NLR) [14], and hyperfibrinogenemia [15], among others.

In recent years, increasing attention has been paid to the impact of nutritional and immune status on cancer prognosis. The Prognostic Nutritional Index (PNI), calculated using serum albumin concentration and peripheral blood lymphocyte count, serves as a practical marker reflecting both aspects [16]. As a simple, inexpensive, and routinely accessible tool, PNI has demonstrated prognostic significance in various solid tumors and hematologic malignancies, including colorectal, gastric, and lung cancers, as well as lymphoma [17,18,19,20,21]. Its ease of use and reliance on standard laboratory tests make it especially suitable for low-risk MDS, where extensive molecular profiling may not be routinely available. However, evidence supporting its prognostic value specifically in low-risk MDS patients remains limited.

Systemic oxidative stress (SOS), defined as an imbalance between free radicals and antioxidant mechanisms, has been associated with cancer development, progression, metastasis, and treatment resistance [22]. Reactive oxygen species (ROS) influence various intracellular signaling pathways including those regulating proliferation, differentiation, and apoptosis [22,23]. In hematological malignancies, particularly in MDS, elevated ROS levels and decreased glutathione concentrations have been observed [24,25]. These redox imbalances are also considered key contributors to drug resistance [26].

The SOS score is calculated using five routine laboratory biomarkers: serum creatinine, albumin, total bilirubin, lactate dehydrogenase (LDH), and blood urea nitrogen (BUN). While the prognostic value of SOS has been previously demonstrated in several solid tumors such as breast, lung, and gastric cancers [23,24,25], its role in MDS has not been previously evaluated. To the best of our knowledge, this is the first study to investigate the prognostic impact of the SOS score in patients with low-risk MDS, offering a novel contribution to the understanding of oxidative stress in this disease context.

This study aimed to identify novel prognostic markers and assess the impact of inflammation, nutritional status, and oxidative stress measured at the time of diagnosis on survival outcomes in patients with low-risk MDS. By focusing exclusively on this specific subgroup, the study provides novel insights into how accessible markers such as the PNI and the SOS score may complement and potentially enhance traditional prognostic systems like IPSS and R-IPSS.

## 2. Materials and Methods

This retrospective study included 175 low-risk MDS patients diagnosed between January 2008 and December 2022 at the Adult Hematology Department of Uludağ University, Bursa. These patients were retrospectively analyzed using follow-up data available through January 2024. All clinical, hematological, biochemical, and genetic data were obtained retrospectively from hospital records and laboratory information systems at the time of diagnosis. All laboratory parameters were analyzed using standard automated techniques as part of routine care. At the time of diagnosis, data were collected on leukocytes (WBCs), hemoglobin, mean corpuscular volume (MCV), platelets, neutrophils, lymphocytes, monocytes, red cell distribution width (RDW), platelet distribution width (PDW), ferritin, creatinine, BUN, glomerular filtration rate (GFR), LDH, albumin, total protein, cholesterol, and total bilirubin levels. The following indices were calculated: PNI [serum albumin (g/L) + 5 × lymphocyte count (×10^9^/L)], NLR (neutrophil/lymphocyte), Lymphocyte-to-Monocyte Ratio (LMR) (lymphocyte/monocyte), PLR (platelet/lymphocyte), Systemic Inflammatory Response Index (SIRI) (neutrophil × monocytes/lymphocytes), Systemic Immune Index (SII) (neutrophil × platelet/lymphocyte), NSII (Novel Systemic Immune–Inflammation Index) (lymphocyte × platelet/neutrophil), SOS (−0.64 × creatinine + 0.56 × total bilirubin + 0.86 × LDH + 0.70 × BUN − 0.68 × albumin), Pan-Immune Inflammation Value (PIV) (platelet × monocyte × neutrophil/lymphocyte), Ferritin-to-Albumin Ratio (FAR), and Naples Prognostic Score (NPS). Patients were classified into subtypes according to the World Health Organization (WHO) 2016 classification [hypocellular MDS, MDS with 5q syndrome, MDS with single-lineage dysplasia (MDS-SLD), MDS with multilineage dysplasia (MDS-MLD), MDS with ring sideroblasts (MDS-RS), MDS with excess blasts-1 (MDS-EB1), MDS with excess blasts-2 (MDS-EB2), MDS unclassified (MDS-U)] and into risk groups according to IPSS and R-IPSS. High-risk patients (intermediate-2 and high-risk according to IPSS, high and very high-risk according to R-IPSS) were not included in the study.

The primary endpoint of the study was OS, defined as the duration from diagnosis to death from any cause or until the last clinical follow-up.

Methodology of Cross-Validation Analysis: A 5-fold cross-validation approach was applied to the dataset. The following steps were taken to ensure rigorous validation.

### 2.1. Data Partitioning

The dataset, consisting of 175 units, had an overall mortality rate of 66.3%.

To preserve this distribution, the dataset was randomly divided into five equal subsets (folds), ensuring that each fold maintained a similar proportion of mortality cases and non-events.

As a result, the mortality rates in each fold were 67.6%, 65.7%, 66.7%, 65.7%, and 65.7%, respectively.

### 2.2. Model Training and Validation

Four folds were used for each iteration as the training dataset, while the remaining fold served as the test dataset.

Cox proportional hazards regression models were trained on the training folds using backward stepwise selection to determine the final predictors.

To ensure consistency, the same set of predictors included in the main study’s univariate analysis was used in each fold.

Following multivariate analysis, only the variables that remained significant in the final model were used to calculate XBeta values for the test set using their respective regression coefficients.

The test fold was then used to evaluate model performance.

### 2.3. Performance Evaluation

The predictive performance was assessed using the area under the receiver operating characteristic curve.

Area Under the Curve (AUC) values were calculated separately for each test fold, and the overall performance was summarized as mean AUC ± standard deviation (SD).

### 2.4. Statistical Interpretation

The AUC values for each fold, along with their 95% confidence intervals (CIs) and *p*-values. The overall model performance was reported as AUC = 0.71 ± 0.12, suggesting a moderate predictive capability.

### 2.5. Statistical Analysis

The statistical analyses were conducted using IBM SPSS Version 25.0 Statistics for Windows, (Statistical Package for the Social Sciences, IBM Corp., Armonk, NY, USA). Descriptive statistics are presented as *n* and % for categorical variables, mean ± SD and median (min–max) for continuous variables. The results of ROC analyses for the prediction of mortality and transformation to AML by various clinical parameters are given. The appropriate cut-off value was determined according to the maximum Youden index. The Kaplan–Meier method was used to compare survival times between various clinical parameter groups. Finally, univariate and multivariate Cox regression analysis Backward LR method was performed. In the context of multivariate analysis, parameters with a *p*-value of less than 0.05 in univariate analysis were incorporated. A *p*-value of less than 0.05 was considered statistically significant. Multicollinearity was assessed using the variance inflation factor (VIF); variables with VIF > 5 were considered collinear and were excluded from the final multivariate model.

## 3. Results

### 3.1. Patients’ Characteristics

Among the 175 low-risk MDS patients included in the study, 89 (50.9%) were classified as MDS with multilineage dysplasia (MDS-MLD), 59 (33.7%) as MDS with single-lineage dysplasia (MDS-SLD), 15 (8.6%) as MDS with excess blasts-1 (MDS-EB-1), 6 (3.4%) as hypocellular MDS, and 6 (3.4%) as MDS with 5q syndrome. The median age of the patients was 68 years, with a range of 29 to 88 years. The cohort consisted of 88 women (50.3%) and 87 men (49.7%). According to the IPSS score, 87 patients (49.7%) were classified in the low-risk group, and 88 patients (50.3%) were classified in the intermediate-1 risk group. A subsequent analysis of the patients’ genetic profiles revealed that the majority (87.5%) exhibited normal karyotypes. The treatments received by the patients were also analyzed, revealing that 70.3% of the patients received ESA, 13.8% received only transfusion, 10.3% received azacitidine, 3.4% received lenalidomide, and 2.2% received immunosuppressive treatment. The median OS for the patients was 63.1 months, with a 95% confidence interval (CI) of 49.4 to 76.7 months. During the follow-up period, 9 patients (5.1%) progressed to AML. At the end of the study, 59 patients (33.7%) were still alive, while 116 patients (66.3%) had died. The demographic characteristics of the patients are summarized in Table 1.

### 3.2. Nutrition-Based and Inflammation Scores Analysis

The cut-off value for the PNI was established at 47.47, representing the highest Youden index on the ROC curve, which indicates the optimal balance between sensitivity and specificity. ROC analysis result table is shown in Table 2. Based on this cut-off, patients were classified into two groups: those with a low PNI (≤47.47) and those with a high PNI (>47.47). The low-PNI group comprised 92 patients, while the high-PNI group comprised 83 patients. When comparing the two groups, the low-PNI group exhibited higher R-IPSS scores (*p* = 0.013) and lower levels of leukocytes (*p* < 0.001), lymphocytes (*p* < 0.001), hemoglobin (*p* = 0.029), MCV (*p* = 0.020), platelets (*p* < 0.001), albumin (*p* < 0.001), total protein (*p* < 0.001), NSII (*p* < 0.001), and LMR (*p* < 0.001). Additionally, this group showed higher levels of creatinine (*p* = 0.007), LDH-to-albumin ratio (*p* < 0.001), LDH-to-lymphocyte ratio (*p* < 0.001), SIRI (*p* = 0.005), PLR (*p* = 0.029), NLR (*p* < 0.001), and SOS (*p* = 0.038). The low-PNI group was also associated with a lower overall survival (OS) (*p* < 0.001). Clinical and laboratory characteristics of the two groups and all patients are summarized in Table 3.

The PNI prediction was not found to be statistically significant in terms of its ability to predict conversion to AML (*p* = 0.668) (Table 4).

### 3.3. Univariate and Multivariate Analysis of Nutrition-Based and Inflammation Scores

In the univariate analysis, the following factors were identified as statistically significant predictors of poor prognosis: age, gender, IPSS and R-IPSS scores, transfusion dependency, AML transformation, leukocyte count, lymphocyte count, hemoglobin level, MCV, platelet count, LDH, albumin, total protein, ferritin, NLR, SIRI, SOS, PNI, the LDH-to-albumin ratio, and the LDH-to-lymphocyte ratio. In the univariate analysis, there was no statistically significant association between treatment modality [hypomethylating agents (HMAs) and other treatments] and prognosis (HR 0.911, 95% CI 0.511-1.624, *p* = 0.752).

In the multivariate analysis, age, gender, R-IPSS, AML transformation, PNI score, and SOS score were identified as independent risk factors for OS in low-risk MDS patients. Detailed results of the univariate and multivariate data analyses are summarized in Table 5. Advanced age (HR 1.057, 95% CI 1.036–1.078, *p* < 0.001), male gender (HR 0.529, 95% CI 0.359–0.779, *p* = 0.001), high R-IPSS (HR 1.532, 95% CI 1.242–1.889, *p* < 0.001), low PNI (HR 0.954, 7 > 95%CI 0.931–0.978, *p* < 0.001), high SOS (HR 1.003, 95%CI 1.001–1.006, *p* = 0.002), and AML transformation (HR 6.381, 95% CI 2.966–13.727, *p* < 0.001) were associated with poor prognosis.

### 3.4. Multivariate Analysis of Nutrition-Based and Inflammation Scores

In the multivariate analysis, age, gender, R-IPSS, AML transformation, PNI score, and SOS score were identified as independent risk factors for OS in patients with low-risk MDS. Detailed results of the univariate and multivariate data analyses are summarized in Table 5. Advanced age (HR = 1.055, 95% CI: 1.034–1.076, *p* < 0.001), male gender (HR = 0.577, 95% CI: 0.394–0.844, *p* = 0.005), high R-IPSS score (HR = 1.554, 95% CI: 1.266–1.908, *p* < 0.001), low PNI score (HR = 1.598, 95% CI: 1.076–2.372, *p* = 0.02), high SOS score (HR = 1.003, 95% CI: 1.001–1.006, *p* = 0.002), and AML transformation (HR = 5.509, 95% CI: 2.600–11.672, *p* < 0.001) were independently associated with poor prognosis.

### 3.5. Survival Analysis

**Figure 1:**
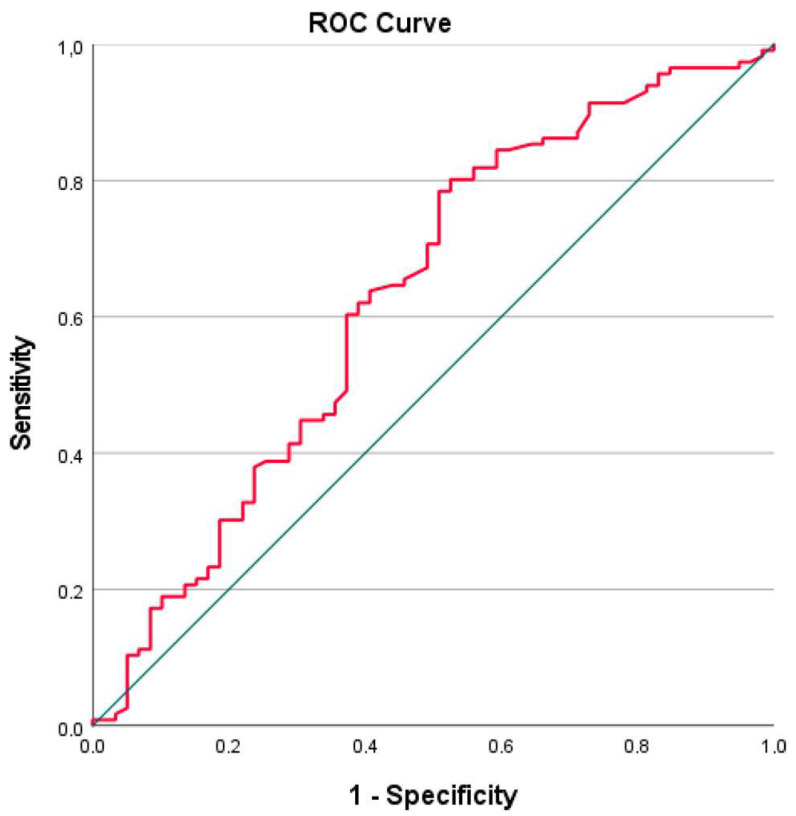
ROC curve of PNI levels for predicting mortality.

**Figure 2:**
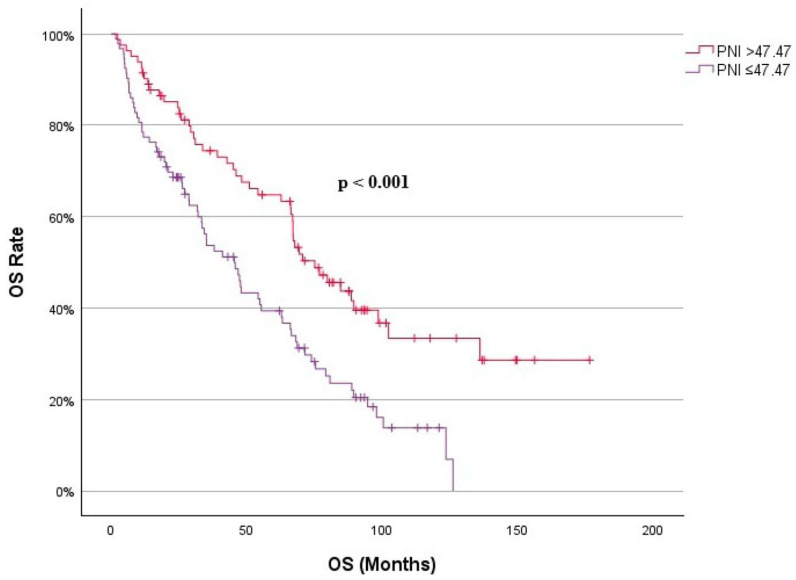
Overall survival by PNI, Kaplan–Meier analysis.

The optimal cut-off value for age to predict mortality was found to be 65.5 years. The Area Under the Curve (AUC) for this cut-off was 0.675 (95% CI 0.590–0.761), with a sensitivity of 68% and a specificity of 61% for ages at or below this value. In addition, neutrophil and monocyte counts, RDW, PDW, cholesterol, LMR, PLR, SII, NSII, creatinine, BUN, GFR, total bilirubin, LDH–ferritin ratio, albumin–NLR ratio, neutrophil/albumin ratio, PIV, FAR, and NPS did not show a significant statistical impact on prognosis. ROC analysis determined the optimal cut-off value for PNI to distinguish mortality as 47.47. The AUC was 0.629 (95% CI 0.538–0.720), with a sensitivity of 60% and specificity of 63% for values at or below this cut-off (see Figure 1). Kaplan–Meier survival analysis comparing low-PNI and high-PNI groups showed that the OS was significantly lower in the low-PNI group compared with the high-PNI group (45.46 months vs. 75.1 months, *p* < 0.001, see Figure 2). To further evaluate the prognostic performance of PNI, additional cut-off values were assessed for their sensitivity and specificity in predicting mortality (Table 2). Higher cut-offs (e.g., 55.0 or 60.0) increased sensitivity but reduced specificity, while lower thresholds (e.g., 44.0 or 40.0) enhanced specificity at the expense of sensitivity. Among the evaluated values, a cut-off of 47.47 provided the most balanced diagnostic performance and the clearest survival separation, confirming it as the most clinically relevant threshold.

In terms of survival outcomes, 47.47 yielded the greatest OS difference (45.46 vs. 75.1 months, *p* < 0.001), whereas lower thresholds such as 44.0 and 40.0 showed smaller yet significant survival differences (*p* = 0.002 and *p* = 0.006, respectively). Conversely, higher cut-offs like 55.0 and 60.0 produced either borderline or nonsignificant results (*p* = 0.040 and *p* = 0.461, respectively), further supporting the use of 47.47.

In multivariate analysis, patients with a low PNI had a significantly higher risk of mortality (HR = 1.598, 95% CI: 1.076–2.372, *p* = 0.020), while no significant association was observed between PNI and AML transformation (*p* = 0.668), suggesting its prognostic value is limited to OS rather than leukemic progression.

The results of the cross-validation analysis are presented in Table 6. These findings indicate that the model’s predictive power remains relatively consistent across different test sets with AUC values ranging between 0.55 and 0.85. While Fold 3 and Fold 5 yielded nonsignificant *p*-values (*p* > 0.05), the overall performance (mean AUC = 0.71 ± 0.12) suggests that the model maintains a reasonable level of discrimination.

## 4. Discussion

This study represents the largest cohort to date evaluating the prognostic value of the PNI and is the first to investigate the clinical relevance of the SOS score in patients with low-risk MDS. A low PNI value was significantly associated with poor prognosis, and PNI at diagnosis was identified as an independent risk factor for overall survival in these patients. Several biological pathways may explain the association between low PNI and increased mortality in MDS. Hypoalbuminemia, as a component of PNI, reflects systemic inflammation, poor nutritional status, or hepatic dysfunction. Lymphopenia, another component, suggests immune suppression and decreased host defense. Both factors contribute to poor treatment tolerance, increased susceptibility to infections, and reduced physiological resilience, all of which may negatively impact survival in patients with MDS.

These findings are important because most clinicians currently manage low-risk MDS patients, especially those with low R-IPSS, without treatment until disease progression (increasing blasts, progressive pancytopenia, or transfusion dependence). Survival in low-risk MDS patients ranges from 2.4 to 11.8 years, depending on age. However, without advanced molecular testing such as next-generation sequencing (NGS), clinical evaluation may remain incomplete. According to our study, comparison of low-PNI and high-PNI groups showed that OS was significantly lower in the low-PNI group compared with the high-PNI group (45.46 months vs. 75.1 months, *p* < 0.001).

Numerous studies have suggested that inflammation and nutrition can serve as prognostic indicators in cancer patients [20,27,28]. Additionally, recent research supports the activation of inflammatory pathways in the bone marrow of low-risk MDS patients [29,30,31].

The PNI reflects the interactions between host immunity as indicated by lymphocyte count, tumor-induced inflammation, and nutritional status. Several studies have proposed that PNI can predict prognosis across various malignancies [20,27,32,33]. However, whether PNI is an effective nutritional and immunological marker for predicting prognosis in low-risk MDS remains unclear.

In a recent study involving 121 MDS patients, the high-PNI cohort demonstrated significantly better OS (*p* = 0.020), with an optimal cut-off value of 44. Subgroup analysis revealed that the impact of PNI on outcomes was most pronounced in intermediate-risk patients [34].

Similarly, in this study, the low-PNI group was associated with poorer OS, with an optimal cut-off value of 47.47. For this cut-off, the sensitivity was 60%, and the specificity was 63%.

Given its ease of calculation and accessibility, PNI may be integrated into routine assessments at diagnosis, especially for patients classified as low-risk by IPSS/R-IPSS, to help identify individuals who may benefit from early intervention or closer follow-up. From a clinical perspective, these results highlight that even patients categorized as low-risk may experience substantially different outcomes based on nutritional and oxidative stress markers.

Notably, PNI did not show a statistically significant association with AML transformation in our analysis. This distinction highlights that overall health-related frailty, rather than disease biology, may play a dominant role in determining non-leukemic mortality in low-risk MDS patients. This finding supports the need to distinguish between overall mortality risk and transformation-specific risk in MDS.

Excessive oxidative stress can cause DNA damage and genomic instability, leading to a loss of cellular integrity, function, and viability [22]. The prognostic significance of SOS has been studied in various cancers, including breast, lung, and gastric cancers, where it has been identified as an independent risk factor [23,24,25]. To the best of our knowledge, this study is the first to establish a relationship between SOS and MDS prognosis. The study found that patients with high SOS had worse survival outcomes.

Clonal hematopoiesis not only raises the risk of cancer and mortality but also increases the likelihood of cardiovascular disease, particularly due to mutations in genes such as ten-eleven translocation 2 (TET2), DNA methyltransferase 3 alpha (DNMT3A), ASXL transcriptional regulator 1 (ASXL1), and Janus kinase 2 (JAK2). Although the role of inflammatory signaling in the relationship between clonal hematopoiesis and aging is not fully understood, chronic age-related inflammation likely influences hematopoietic stem cells, contributing to both cardiovascular disease and immune responses [35].

In our cohort, after excluding deaths from AML transformation, secondary malignancies, infection, and bleeding, cardiovascular causes accounted for 54.3% of all deaths, a rate notably higher than the 30–40% typically reported in individuals over 60 years of age. Given that MDS predominantly affects an elderly population, pre-existing cardiovascular conditions may worsen due to inflammation. These findings suggest that early cardiovascular assessment and close monitoring at diagnosis may help reduce the risk of cardiovascular-related mortality.

In our cohort, several markers that have been evaluated in different studies, including neutrophil and monocyte counts, RDW, PDW, cholesterol, NLR, PLR, SII, NSII, creatinine, BUN, GFR, total bilirubin, LDH–ferritin ratio, albumin–NLR ratio, neutrophil/albumin ratio, PIV, FAR, and NPS, did not show a significant statistical impact on the prognosis of MDS. However, this discrepancy may be attributed to several factors, including the smaller sample size in our study, the higher median age of patients in some reports [11], the greater proportion of higher-risk cases according to IPSS and R-IPSS classifications in other cohorts [12], or the inclusion of CMML and CMML-like patients in those studies [9,11].

Our study has several limitations. First, it was retrospective in design and conducted at a single center. Second, the selected inflammatory markers may not comprehensively capture the complexity of the inflammatory response. Third, molecular profiling, including next-generation sequencing (NGS), was not routinely performed. Lastly, the lack of external validation or an independent cohort limits the generalizability of our findings. Future prospective, multicenter studies are needed to confirm these results.

## 5. Conclusions

This study demonstrates that both the PNI and the SOS score are independent predictors of OS in patients with low-risk MDS. These are accessible, cost-effective, and routinely measurable parameters that reflect nutritional status, immune competence, systemic inflammation, and oxidative stress burden at diagnosis. Their integration into clinical practice may improve risk stratification, especially in settings where molecular diagnostics are unavailable.

## 6. Recommendations

Based on the current evidence, the PNI and SOS scores should be further evaluated as complementary components of existing prognostic models. These findings suggest that anti-inflammatory or antioxidant therapies may play a role in slowing disease progression in MDS. Prospective, multicenter studies are warranted to validate their generalizability and support their integration into routine clinical practice.

## Figures and Tables

**Table 1:** Demographic and clinical characteristics of the patients.

Variables	*n* = 175 (%)
**Age (years)**	
Median (min–max)	68 (29–88)
*≤60*	50 (28.6)
*61–70*	62 (35.4)
*>70*	63 (36.0)
**Gender**	
*Male*	87 (49.7)
*Female*	88 (50.3)
**MDS subtypes**	
*Hypocellular MDS*	6 (3.4)
*MDS-5Q syndrome*	6 (3.4)
*MDS-EB1*	15 (8.6)
*MDS-MLD*	89 (50.9)
*MDS-SLD*	59 (33.7)
**Cytogenetic profile**	
*Del (5q)*	6 (3.4)
*Del (20q)*	3 (1.8)
*Del (11q)*	4 (2.2)
*Del (7q)*	4 (2.2)
*Trisomy 8*	5 (2.9)
*Normal Karyotype*	153 (87.5)
**IPSS score**	
*Median (min–max)*	0.5 (0–1)
*Low*	87 (49.7)
*Intermediate-1*	88 (50.3)
**R-IPSS score**	
*Median (min–max)*	2.5 (0–4.5)
*Very low*	28 (16)
*Low*	113 (64.6)
*Intermediate*	34 (19.4)
**Transfusion dependence**	
*None*	108 (61.7)
*Present*	67 (38.3)
**Treatment modality**	
*ESA*	123 (70.3)
*Lenalidomide*	6 (3.4)
*Azacitidine*	18 (10.3)
*IST*	4 (2.2)
*Transfusion Only*	24 (13.8)
**AML conversion**	
*None*	166 (94.9)
*Present*	9 (5.1)
**Mortality**	
*Alive*	59 (33.7)
*Deceased*	116 (66.3)
**Cardiovascular death**	
*None*	53 (45.7)
*Present*	63 (54.3)

Abbreviations: MDS: Myelodysplastic Syndrome; MDS-EB1: MDS with excess blasts-1; MDS-MLD: MDS with multilineage dysplasia; MDS-SLD: MDS with single-lineage dysplasia MDS; AML: acute myeloid leukemia; IPSS: International Prognostic Scoring System; R-IPSS: Revised International Prognostic Scoring System; Del: deletion; ESA: Erythropoiesis-stimulating agent; IST: immunosuppressive therapy.

**Table 2:** Analysis of the predictive value of PNI in distinguishing mortality.

	AUC	95% CI	Cut-Off	Sensitivity	Specificity	*p*-Value
PNI	0.629	0.538–0.720	47.47	60	63	**0.005**
PNI	0.629	0.538–0.720	44	37.07	76.27	**0.005**

AUC: Area Under the Curve; 95% CI: confidence interval.

**Table 3:** Clinical and laboratory characteristics of the patients.

Variables, Median (Min–Max)	PNI ≤ 47.47 (*n* = 92)	PNI > 47.47 (*n* = 83)	Total (*n* = 175)	*p*
**Gender**				
*Female*	44	44	88	0.493 ^x2^
*Male*	48	39	87	
**Age (years)**	69 (31–88)	66 (29–83)	68 (29–88)	0.052 ^m^
**MDS subtype**				
*Hypoplastic MDS*	6	0	6	
*5-Q syndrome*	2	4	6	
*MDS-EB-1*	9	6	15	0.139 ^x2^
*MDS-SLD*	46	43	89	
*MDS-MLD*	29	30	59	
**Leukocytes, /µL**	3895 (500–12,000)	4950 (2200–10,800)	4560 (500–12,000)	**<0.001 ^m^**
**Lymphocytes, /µL**	955 (250–2510)	1890 (862–5100)	1400 (250–5100)	**<0.001 ^m^**
**Monocytes, /µL**	349.5 (20–4300)	452 (26–1190)	415 (20–4300)	0.154 ^m^
**Neutrophils, /µL**	2355 (330–10,000)	2450 (150–8350)	2400 (150–10,000)	0.893 ^m^
**Hemoglobin, g/dL**	8.75 ± 1.58	9.27 ± 1.49	9 (4.0–12.8)	**0.029 ^t^**
**MCV, fL**	91.5 (64–117)	96 (62–120)	93 (62–120)	**0.020 ^m^**
**Platelets, ×10^9^**	136 (5.3–457)	233 (5.9–714)	165 (5.9–714)	**<0.001 ^m^**
**IPSS**	0.5 (0–1)	0 (0–1)	0.5 (0–1)	0.109 ^m^
**R-IPSS**	2.5 (1–4.5)	2 (0–4.5)	2.5 (0–4.5)	**0.013 ^m^**
**Transfusion dependence**				
*None*	54	54	108	0.387 ^x2^
*Present*	38	29	67	
**Ferritin, µg/L**	292 (5–6800)	312 (4.5–2412)	297 (4.5–6800)	0.948 ^m^
**LDH, U/L**	223 (114–983)	202 (114–612)	205 (114–983)	0.232 ^m^
**Albumin, g/L**	37 (10–43)	43 (35–49)	40 (10–49)	**<0.001 ^m^**
**Total protein, g/L**	68 (10–84)	72 (61–85)	70 (10–85)	**<0.001 ^m^**
**Cholesterol, mg/dL**	143 (85–363)	161 (97–275)	158 (85–363)	0.117 ^m^
**Total bilirubin, mg/d**	0.67 (0.15–5.2)	0.66 (0.12–2.65)	0.66 (0.12–5.2)	0.815 ^m^
**Creatinine, mg/dL**	0.91 (0.5–7)	0.80 (0.5–1.72)	0.84 (0.5–7)	**0.007 ^m^**
**BUN, mg/dL**	21 (7–81)	18 (9–45)	20 (7–81)	**0.006 ^m^**
**LDH/albumin**	6.22 (0–44.68)	4.72 (0–15.3)	5.26 (0–44.68)	**<0.001 ^m^**
**LDH/lymphocytes**	0.22 (0–1.89)	0.11 (0–0.35)	0.14 (0–1.89)	**<0.001 ^m^**
**SIRI**	0.92 (0.03–18.5)	0.54 (0–4.07)	0.68 (0–18.5)	**0.005 ^m^**
**NSII**	51.0 (1.2–612)	156 (11.2–3360)	81.4 (1.2–3360)	**<0.001 ^m^**
**SII**	326.5 (20.2–2935)	248.8 (2.11–2700)	297.5 (2.11–2935)	0.201 ^m^
**LMR**	2.97 (0.06–38.5)	4.49 (1.36–160)	3.71 (0.06–160)	**<0.001 ^m^**
**PLR**	132.7 (5.1–808)	105.3 (2.11–432)	118.6 (2.11–808)	**0.029 ^m^**
**NLR**	2.82 (0.37–15.6)	1.39 (0.03–4.99)	1.83 (0.03–15.6)	**<0.001 ^m^**
**SOS**	184.8 (102–847.7)	157 (79.6–514)	168 (79.6–847.7)	**0.038 ^m^**
**AML Transformation**				
*Present*	5	4	9	0.854 ^x2^
*None*	87	79	166	
**Final status**				
*Alive*	22	37	59	**0.004 ^x2^**
*Deceased*	70	46	116	
**OS (months)**	45.46 (33.2–57.6)	75.1 (56.6–93.5)	63.1 (49.4–76.7)	**<0.001 ^k^**

Abbreviations: MDS: Myelodysplastic Syndrome; MDS-EB-1: MDS with increased blasts-1; MDS-MLD: MDS with multilineage dysplasia; MDS-SLD: MDS with single-lineage dysplasia; MCV: mean corpuscular volume; IPSS: International Prognostic Scoring System; R-IPSS: Revised International Prognostic Scoring System; LDH: lactate dehydrogenase; BUN: Blood urea nitrogen; SIRI: Systemic Inflammatory Response Index; NSII: Novel Systemic Immune–Inflammation Index; SII: Systemic Immune Index; LMR: Lymphocyte monocyte ratio; PLR: Platelets lymphocyte ratio; NLR: Neutrophil lymphocyte ratio; AML: acute myeloid leukemia; SOS: systemic oxidative stress score; OS: overall survival; ^m^: Mann–Whitney U; ^x2^: Pearson Chi-square; ^k^: Kaplan–Meier Survival Analysis; ^t^ Independent Samples *t*-test; Bold *p* < 0.05 considered statistically significant.

**Table 4:** Analysis of the predictive value of PNI in distinguishing AML.

	AUC	95% CI	Cut-Off	Sensitivity	Specificity	*p*-Value
PNI	0.543	0.395–0.690	47.14	55	51	0.668

AUC: Area Under the Curve; 95% CI: confidence interval, PNI: Prognostic nutritional index.

**Table 5:** Univariate and multivariate prognostic data analysis for OS.

	Univariate Cox Regression			Multivariate Cox Regression		
Risk Factors	HR	95% CI	*p*	HR	95% CI	*p*-value
**Age**	**1.046**	**1.027–1.066**	**<0.001**	**1.057**	**1.036–1.078**	**<0.001**
**Gender**	**0.575**	**0.397–0.832**	**0.003**	**0.529**	**0.359–0.779**	**0.001**
IPSS	3.020	1.710–5.333	<0.001			
**R-IPSS**	**1.469**	**1.215–1.777**	**<0.001**	**1.532**	**1.242–1.889**	**<0.001**
Transfusion dependence	2.101	1.456–3.031	<0.001			
Treatment Modality (HMA vs. Other)	0.911	0.511–1.624	0.752			
**AML Transformation**	**3.265**	**1.623–6.568**	**<0.001**	**6.381**	**2.966–13.727**	**<0.001**
Leukocyte, /µL	1.000	1.000–1.000	0.032			
Lymphocyte, /µL	1.000	0.999–1.000	0.011			
Hemoglobin, g/dL	0.846	0.753–0.950	0.005			
MCV, fL	1.016	1.000–1.033	0.049			
Platelet, ×10^9^	1.000	1.000–1.000	0.018			
LDH, U/L	1.002	1.000–1.004	0.026			
Albumin, g/L	0.952	0.926–0.979	<0.001			
Ferritin, µg/L	1.000	1.000–1.000	0.024			
**PNI**	**0.957**	**0.937–0.979**	**<0.001**	**0.954**	**0.931–0.978**	**<0.001**
NLR	1.085	1.000–1.177	0.049			
SIRI	1.087	1.015–1.164	0.016			
**SOS**	**1.003**	**1.001–1.005**	**0.009**	**1.003**	**1.001–1.006**	**0.002**
LDH/Lymphocytes	2.277	1.259–4.118	0.007			
LDH/Albumin	1.076	1.031–1.123	<0.001			

Abbreviations: IPSS: International Prognostic Scoring System; R-IPSS: Revised International Prognostic Scoring System; AML: acute myeloid leukemia; MCV: mean corpuscular volume; LDH: lactate dehydrogenase; PNI: Prognostic Nutritional Index; NLR: neutrophil-to-lymphocyte ratio; SIRI: Systemic Inflammatory Response Index, SOS: systemic oxidative stress score; HMA: hypomethylating agent, HR: hazard ratio, CI: confidence interval.

**Table 6:** A 5-fold cross-validation analysis.

Fold	AUC	95% CI	*p*-Value	Mortality Rate
**Fold 1**	0.76	0.59–0.92	**0.002**	67.6%
**Fold 2**	0.76	0.59–0.94	**0.004**	65.7%
**Fold 3**	0.55	0.34–0.76	0.663	66.7%
**Fold 4**	0.85	0.72–0.98	**<0.001**	65.7%
**Fold 5**	0.63	0.44–0.82	0.181	65.7%
**Overall (Mean ± SD)**	0.71 ± 0.12			**66.3 ± 0.8%**

## Data Availability

The datasets used and/or analyzed during the current study available from the corresponding author on reasonable request.

## Abbreviations

MDS: Myelodysplastic Syndrome, AML: acute myeloid leukemia, IPSS: International Prognostic Scoring System, R-IPSS: Revised International Prognostic Scoring System, IPSS-M: Molecular International Prognostic Scoring System, ALT: alanine aminotransferase, PLR: platelet-to-lymphocyte ratio, NLR: neutrophil-to-lymphocyte ratio, PNI: Prognostic Nutritional Index, SOS: systemic oxidative stress, ROS: reactive oxygen species, LDH: lactate dehydrogenase, BUN: blood urea nitrogen, WBCs: leukocytes, MCV: mean corpuscular volume, RDW: red cell distribution width, PDW: platelet distribution width, GFR: glomerular filtration rate, LMR: Lymphocyte-to-Monocyte Ratio, SIRI: Systemic Inflammatory Response Index, SII: Systemic Immune Index, NSII: Novel Systemic Immune–Inflammation Index, PIV: Pan-Immune Inflammation Value, FAR: Ferritin-to-Albumin Ratio, NPS: Naples Prognostic Score, WHO: World Health Organization, MDS-SLD: MDS with single-lineage dysplasia, MDS-MLD: MDS with multilineage dysplasia, MDS-RS: MDS with ring sideroblasts, MDS-EB1: MDS with excess blasts-1, MDS-EB2: MDS with excess blasts-2, MDS-U: MDS unclassified, AUC: Area Under the Curve, SD: standard deviation, CI: confidence interval, OS: overall survival, ESAs: Erythropoiesis-stimulating agents, Del: deletion, IST: immunosuppressive therapy, HMAs: hypomethylating agents, NGS: next-generation sequencing, HR: hazard ratio, TET2: ten-eleven translocation 2, DNMT3A: DNA methyltransferase 3 alpha, ASXL1: ASXL transcriptional regulator 1, JAK2: Janus kinase 2.
